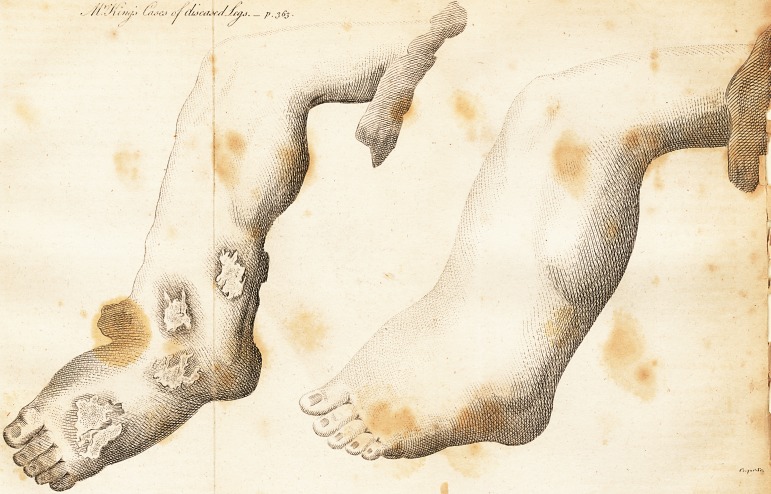# Mr. King's Case of Elephantiasis

**Published:** 1806-10

**Authors:** John King

**Affiliations:** South Carolina


### *To the Editors of the Medical and Physical Journal.*

GENTLEMEN,

I Avail myself of the occasion of forwarding you, through the hands of Samuel Chilver, Esq. Delineations of two Legs, which I think very curious in respect of their figure.

Figure 1 is denominated Elephantiasis.

Figure 2 I designate by the appropriate term of *Mammothsis* or Mammoth-like leg.* I have never, in very extended

---

* This leg I am certain was three or four feet in circumference.

Mr. King's Cases of diseased Legs. — p. 363.

Cooper Sc.

tended observations, seen parallels of either cases, nor have I met with medical men who have.

I am, &c.

*South Carolina, Jan.* 1, 1806. JOHN KING.

---

Fig. 1, *(vide plate)* represents the leg of a negro man, more than forty years of age, of a constitution impaired by venereal complaints, and of a strumous habit; he had been near sixteen years afflicted with this enlargement, varying in degree according to circumstances. The foot was originally frost-bitten. Inflammation supervened, and the thickness of the integument progressed, till it described the phænomenon in plate No. 1. The inflammation, though at first of a common kind, became afterwards combined with syphilitic venom, which finding its way to the lymphatics of the member, set up a permanent action of a specific kind.

All the common instruments of medical chirurgery were applied and re-applied in vain for a long term of years; I hoped to produce some change by the bracing plasters long continued; they did indeed operate a better effect than any former topical application. The ulcers, which had used to be a drain on the constitution, nearly healed, but re-appeared with the least stress on the limb in walking.

The white spots exhibit ulcers in various parts, overflowing with the most fœtid fluid, and corroding the soft parts so rapidly as to threaten the total demolition of the foot. At this time the patient's habits, infected from the absorption, of so acrimonious a fluid, suffered greatly, and doubtless would have destroyed the digestive faculty, had the limb been permitted to remain on; for though the patient laboured under venereal complaints, and had a gonorrhœa at the time, I judged it expedient that the limb should be very soon amputated, as I am certain, had it not been done, he would soon have died hectic.

The day preceding the operation, I let blood, and ordered an active purgative, which in some degree diminished the superior part of the leg, but nevertheless I had to pass the knife through swollen parts, but confided in the reduction of what remained to an extensive suppuration. After a few days of sedative means, the patient drank daily two or three pints of a strong decoction of the china, shumach, and sweet bay roots, much esteemed by Negroes as as a powerful antisyphilitic; its good effect on the habit was soon visible, and the cure proceeded as well as could be wished, and much better than could be expected; for as I before said, he was scrophulous by nature; he had albuminous eyes from scrophulous ophthalmia, (which a few months ago I relieved by dividing the blood-vessels all round the transparent cornea, and by other means) the strumous lip, and leaden aspect; was peculiarly indolent, and afflicted with pain on the slightest change of weather, a more sure guide than Fahrenheit; he had fungous gums and pestiferous breath, enlarged and lengthened teeth from scorbutic depravity, loosened from alveolar destruction, presenting a most gloomy countenance; a scaly desquamating skin, ragged, and truly elephantic.

After the operation, I made an incision through the whole extent of the part of the limb which was severed, to satisfy myself on the true state of morbid anatomy. I found the aponeurosis, the tendons, the muscles, the ligaments, the cavity of the ankle-joint, all undiseased; the arteries unossified, the synovia somewhat accumulated, the bones themselves perhaps somewhat enlarged, tho' these two last mentioned circumstances I do not consider peculiar, but as manifested under all occasions of torpor.

It appears evident to me, on a view of the morbid growth of parts, that the disposition of the arteries of the cellular membrane in depositing the matter which constitutes this enlargement, is incontroulable either from an insuperable bias on their side to increase, or through a morbid defect of the absorbents to carry off the *deposita*. For all the cells, including those occupied in anasarca, exhibited on dissection a mixture of parenchyma and fat, and the circle of the cells themselves converted into a very tough membrane.

It only remains to say, that the whole seat of this curious change of figure is cuticular, that it is engendered in the true skin, that a morbid action, set up in the arteries thereof, is probably the exciting cause, for certainly anasarca did not constitute the disease, as when I passed the knife through that bulge above the instep, the skin was three inches thick, and through the whole limb it varied from that to two, one, and half an inch, or thereabouts; all this horrible mischief originated no doubt from local inflammation, afterwards neglected, then combined with syphilis, then irritated by acrid applications, till at length it became ulcerous, most abominably fœtid, and at last incurable, exhibiting with the Negro skin the most genuine elephantiasis.

elephantiasis. No case of amputation (under circumstances of complicated disease) ever proceeded more favourably; and the health, the animal spirits and countenance answered the best expectations.

The figure No. 2, yielded to no medical means whatever, any more than the former, and I am much disposed to consider the two cases as very analogous in their proximate cause, though I have not demonstration of the latter. This case I saw many years ago at Fakenham in Norfolk, (England) when a very young man, and I did not consider the subject much at that time, though I have very often since. Mr. Peter Raven, jun. of Lytchham, Norfolk, was so kind as to introduce me to this case. Mr. Raven, I believe, described it to Mr. Henry Cline, and I think he advised amputation. The astonishing increase in the leg, threatened to invade the thigh, but I imagine the ductility of the parts saved the leg from ulceration. I do not know how the case terminated; I remember the limb was hard, polished, warm, and blushing, and partially desquammatory; its stupendous size made me compare it to a fat hog with a pendent belly, the foot representing the head with the ears cut off.

I have seen most frightful enlargements from anasarca, but never any thing to equal this *cuticular deposit.*

The patient had symptomatic fever and used anodynes; he sat up at times in his bed, was about forty years of age, a publican by profession, and a healthy, well-favoured man.

I do not know the history of this case; but I consider both as diseases of the true skin, as similar in their proximate cause, and as incurable diseases, (whatever cause they may proceed from) if not arrested by art in their incipiency. Whether the operation for the aneurism would put a check to growth, I cannot say, but as to myself, would certainly prefer it to amputation. If the wisdom of our best surgeons, so decided for supposing the fault to be in the absorbents, no physician can affirm such a method would not succeed; and if the matter rested wholly with the arteries, the probability is, that good might be gained, but I leave this hypothesis to the penetration of my countrymen.

As every thing of comparative magnitude on this vast Continent is of the Mammoth kind, I could not refrain from affixing the term *Mammothsis,* or *Mammoth-like* leg, to the figure No. 2. A "Monstrum horrendum informe ingens."

## Figures and Tables

**Figure f1:**